# Migrating populations--a closer view of who, why, and so what.

**DOI:** 10.3201/eid0707.017730

**Published:** 2001

**Authors:** S T Cookson, M Carballo, C M Nolan, J S Keystone, E C Jong

**Affiliations:** Centers for Disease Control and Prevention, Atlanta, Georgia 30333, USA. scookson@cdc.gov


### Migrating Populations—A Closer View of Who, Why, and So What

**Susan T. Cookson,* Manuel Carballo,† Charles M. Nolan,‡ Jay S. Keystone,§ and Elaine C. Jong¶**

*Centers for Disease Control and Prevention, Atlanta, Georgia, USA; †International Centre for Migration and Health, Geneva, Switzerland; ‡Seattle-King County Department of Public Health, Seattle, Washington, USA; §University of Toronto, Toronto, Canada; and ¶University of Washington School of Medicine, Seattle, Washington, USA

In the last decade, human migration increased fourfold; destinations now involve all points on the globe, with religious persecution and political conflict as common reasons to migrate. Environmental disasters and economic factors, causing rapid depletion of natural resources, also impel people to seek job opportunities and an improved standard of living in cities (1).

Since 1985, 36 countries have been involved in conflicts that caused the uprooting of 100 million persons—more than 35 million in 1999 alone (2). These conflicts result in massive displacement of people and communities; increased illness and death; and major social, cultural, ethnic, and material disruption. To mitigate the impact of these detrimental effects exceptional measures need to be taken. However, the number of migrating workers seeking temporary lodging, estimated at 42 million, overshadows the number of people forced to immigrate as a result of conflicts (3). Visitors comprise the largest group of migrating people, with 25 million traveling to Australia, Canada, and the United States annually.

Immigrants bring with them their cultural and health beliefs (4). For instance, Ukrainians believed that positive tuberculin skin tests meant that the bacillus Calmette-Guérin vaccine was effective. However, after resettling in Seattle, they learned that people who test positive have latent tuberculosis. Other societies, for example, Bosnia, teach that pharmaceuticals weaken the body and should be taken only when a person feels ill—making the concept of treating latent infection difficult for Bosnian immigrants to grasp.

Speaking different languages is a substantial barrier to immigrants receiving appropriate health care. In a California school, 1,000 students speak 15 languages. Medical interpreters are usually scarce. Family members are often recruited to translate, but this can lead to misunderstandings. When intervention is available, fear of consequences prevents many immigrants from seeking medical advice and treatment.

Even immigrants securely resettled may be reexposed to diseases when they return home to visit friends and relatives or associate with newly arrived members of their ethnic group. The incidence of malaria and typhoid fever is greater among immigrants returning home for visits than among North American-born travelers (5,6) because the former tend not to obtain pretravel health advice—physicians are not consulted, and sometimes physicians do not provide appropriate advice (7,8). The lack of medical infrastructure in countries of origin and the lack of medical surveillance after resettlement are additional problems.

Current national and international immigration policies are insufficient, in part because conflicting social, political, and health issues impede progress. Public health policies should be more flexible and proactive. Greater understanding is needed regarding the dynamics of human migration and its long-term health consequences. Solutions for development aid and conflict prevention and resolution must be found; health problems must be mitigated through timely responses. As public health officials, we must promote an international vision of migration as an inevitable phenomenon that is key to economic development. Health policies and development aid to mitigate the adverse effects of migration are essential.

### References

1. Castles S, Miller MJ. The age of migration: international population movements in the modern world. New York: Guilford Press; 1998.
2. U.S. Committee for Refugees. World refugee survey 2000. Washington: Immigration and Refugee Services of America, 2000.
3. Parfit M. Human migration. National Geographic 1998;4:6-35.
4. Carballo M, Divino JJ, Zeric D. Migration and health in the European Union. Trop Med Int Health 1998;3:926-44.
5. Steinberg EB, Frisch A, Rossiter S, McClellan J, Ackers M, Mintz ED. Typhoid fever in travelers—who should we vaccinate? Am J Trop Med Hyg 2000;62:158-9.
6. Castelli F, Matteelli A, Caligaris S, Gulletta M, el-Hamad I, Scolari C, et al. Malaria in migrants. Parassitologia 1999;41:261-5.
7. dos Santos CC, Anvar A, Keystone JS, Kain KC. Survey of use of malaria prevention measures by Canadians visiting India. CMAJ 1999;160:195-200.
8. Campbell H. Imported malaria in the UK: advice given by general practitioners to British residents traveling to malaria endemic areas. J R Coll Gen Pract 1987;37:70-2.

Address for correspondence: Susan Cookson, Centers for Disease Control and Prevention, NCID DQ, 1600 Clifton Road, Mailstop E03, Atlanta, Georgia 30333, USA; fax: 404-639-2599; email: scookson@cdc.gov
